# Using Genetics to Understand the Role of Lifestyle and Socioeconomic Factors in the Relationship Between Major Depressive Disorder and Type 2 Diabetes

**DOI:** 10.1016/j.bpsgos.2026.100822

**Published:** 2026-09-09

**Authors:** Renu Bala, Anya Morris, Dale Handley, Francesco Casanova, Harry Green, Mike Elliott, Annie Jeffery, Alexandra C. Gillett, Cathryn M. Lewis, Jessica Tyrrell

**Affiliations:** a Department of Clinical and Biomedical Sciences, Faculty of Health and Life Sciences, University of Exeter, Exeter, United Kingdom; b Social, Genetic and Developmental Psychiatry Centre, Institute of Psychiatry, Psychology and Neuroscience, King’s College London, London, United Kingdom; c Department of Epidemiology and Applied Clinical Research, Division of Psychiatry, University College London, London, United Kingdom; d Department of Medical and Molecular Genetics, Faculty of Life Science and Medicine, King’s College London, London, United Kingdom

**Keywords:** Major depressive disorder, Mendelian randomization, Socio-economic and lifestyle factors, Type 2 diabetes, UK Biobank

## Abstract

**Background:**

Major depressive disorder (MDD) and type 2 diabetes (T2D) are global health problems that frequently co-occur. The bidirectional causal relationship between them is well established, although the potential mediators remain understudied. Here, we investigated the role of lifestyle and socioeconomic traits as mediators of the MDD-T2D relationship using genetic data.

**Methods:**

Using genome-wide association study (GWAS) summary statistics, we tested whether the genetic correlation (*r*_*g*_) between MDD (*n* = 2,000,702) and T2D (*n* = 1,812,017) was influenced by lifestyle (physical activity and sedentary time [*n* = 95,776], cigarettes per day [*n* = 618,489], and drinks per week [*n* = 941,280]) and socioeconomic (educational attainment [*n* = 293,723] and poverty factor [*n* = 453,689]) traits. Mendelian randomization (MR) analyses, using both GWAS and UK Biobank data, were conducted to investigate potential causal relationships. Finally, we applied mediation analysis to quantify the indirect effect of potential mediators.

**Results:**

The strong genetic correlation between MDD and T2D (*r*_*g*_ = 0.184, *p* = 3.5 × 10^−37^) was attenuated when accounting for cigarettes per day (partial *r*_*g*_ = 0.098), physical activity (partial *r*_*g*_ = 0.141), educational attainment (partial *r*_*g*_ = 0.115), and poverty (partial *r*_*g*_ = 0.118). MR demonstrated that a higher genetic liability to MDD and T2D was associated with higher cigarettes per day, poverty, and lower education. MR showed that higher education was associated with lower odds of MDD and T2D, while poverty was associated with higher odds of both conditions. Mediation analysis demonstrated that genetic liability to education and poverty traits substantially mediated the causal relationship in both directions: 28.67% and 24.98% for MDD→T2D and 34.3% and 29.87% for T2D→MDD, respectively.

**Conclusions:**

Our findings highlight the role of socioeconomic traits in the MDD-T2D relationship and demonstrate both the strength and limitations of using MR for investigating complex traits. Further research incorporating gene-environment interactions, policy implications, and diverse populations is needed to better understand this relationship.

## Plain Language Summary

People with depression are more likely to develop type 2 diabetes (T2D) and vice versa. However, it is unclear how these conditions are linked or which risk factors they share. In this study, we used statistical genetics methods and genetic data from large population studies to investigate whether lifestyle and socioeconomic factors may help explain this relationship. We found that education and poverty partially mediated the relationship between depression and T2D in both directions. These findings highlight the importance of addressing social inequalities alongside clinical care to help reduce the burden of both conditions.

Major depressive disorder (MDD) and type 2 diabetes (T2D) are growing global health problems that often co-occur. There is a growing body of evidence that MDD associates with a higher T2D risk and vice versa (1, 2, 3). More recently, work by us and others, using the genetic technique of Mendelian randomization (MR), has suggested bidirectional causal relationships between MDD and T2D (4,5). While we demonstrated the importance of both body mass index and insulin resistance in the MDD-T2D relationship (5), the role of modifiable lifestyle and socioeconomic risk factors in driving this relationship remains unclear.

It is well established that lifestyle factors, including diet, physical activity, smoking, and alcohol intake, are associated with both MDD and T2D. For example, smoking is a key risk factor for both MDD and T2D, with smokers at a 30% to 40% higher risk of T2D (6) and longitudinal evidence showing that smoking is associated with an increased MDD risk (7). Similarly, there is observational evidence that a poor diet and higher alcohol intake predict both T2D and MDD (8, 9, 10). However, the evidence for alcohol remains inconclusive, with a large systematic review suggesting a J-shaped relationship between alcohol and T2D in women (10), and genetic evidence suggesting that MDD associates with alcohol dependence, but alcohol dependence does not associate with MDD (11). Physical inactivity is a well-established T2D risk factor (12), and there is growing evidence supporting the role of physical activity in MDD, including from a recent systematic review demonstrating that exercise is an effective depression treatment (13). We and others have also provided genetic evidence that higher physical activity protects against MDD (14,15).

Socioeconomic factors, including educational attainment and poverty, are important in both MDD and T2D. There is extensive evidence suggesting that lower educational attainment (16, 17, 18) and higher levels of poverty are associated with an increased risk of both conditions (19,20). A recent MR study provided causal evidence that poverty contributes to MDD and schizophrenia (21). Education has been suggested as a key moderator of the MDD-T2D relationship in longitudinal observational analyses (22). However, most studies focus on either MDD or T2D, and those that consider both tend to be small observational studies. Therefore, it is less clear whether socioeconomic factors moderate the bidirectional relationship between MDD and T2D. Additionally, environmental stressors, like socioeconomic and lifestyle factors, indirectly (e.g., parental lifestyle or socioeconomic condition determines access to healthcare, nutritious food, and exposure to healthy lifestyle behaviors early in life) or directly impact an individual’s development either during critical life stages or over the life course, predisposing them to a range of health outcomes (23,24). Therefore, understanding risk factors that may mediate the relationship between MDD and T2D is crucial for appropriate public health messaging and to potentially highlight and introduce new interventions or policy changes to reduce the disease burden.

In this study, we used several complementary genetic approaches to understand the role of 6 lifestyle and socioeconomic traits (physical activity, sedentary time, alcohol [drinks per week], smoking [cigarettes per day], educational attainment, and poverty) in the MDD-T2D relationship. We used genetic correlation (*r*_*g*_) and examined whether adjusting for the genetics of each of the 6 potential mediators partially explains the MDD-T2D relationship. We comprehensively tested the bidirectional relationships between MDD and T2D using a range of MR methods in both genome-wide association study (GWAS) summary statistics and individual-level data from UK Biobank (UKB), to enable sex-stratified analyses. Finally, we used mediation analysis to estimate how much of the MDD-T2D relationship could be explained by lifestyle and socioeconomic factors.

## Methods and Materials

### Data Resources

#### GWAS Summary Statistics: Exposure and Outcome

We used GWAS summary statistics from European ancestry for T2D (1,569,734 controls and 242,283 T2D cases) (25), MDD (1,588,397 controls and 412,305 cases) (26), physical activity and sedentary time (*n* = 95,776) (14), cigarettes per day (*n* = 618,489) (27), drinks per week (*n* = 941,280) (28), educational attainment (*n* = 293,723) (29), and poverty factor (*n* = 453,689) (21). More details can be found in Supplemental Methods.

GWAS summary statistics for MDD, T2D, and the lifestyle/socioeconomic traits were preprocessed using the linkage disequilibrium score regression (LDSC) (30) munging function. All LDSC analyses used LD scores estimated from the 1000 Genomes EUR reference panel.

#### Genetic Correlation and Partial Genetic Covariance and Correlation

We used LDSC to test genetic correlations between MDD and T2D and to understand the shared genetic architecture between them. A positive covariance indicates that genetic variants increasing liability to MDD also tend to increase liability to T2D. Partial LDSC enabled us to assess how this genetic covariance changes when we individually remove the genetic effects of each of the 6 potential mediators, although it does not confer or identify causality or mediation. First, we estimated the genetic correlations between MDD, T2D, and the 6 lifestyle/socioeconomic traits. Then we quantified the contribution of each lifestyle/socioeconomic trait by removing its genetic effects from the MDD-T2D genetic covariance using the *partialLDSC* R package (https://github.com/GEMINI-multimorbidity/partialLDSC), as previously described (31). We also used genomic structural equation modeling (SEM) (32) as a sensitivity analysis to test whether these potential mediators play any role in the MDD-T2D relationship (Supplemental Methods).

Lifestyle/socioeconomic traits that significantly contribute to the MDD-T2D genetic correlation were taken forward for MR analyses.

#### Mendelian Randomization

To better understand the causal relationships between the lifestyle/socioeconomic factors (identified through partial LDSC) and MDD and T2D, we performed 2-sample univariable MR (UVMR), a causal inference method using genetic variants as instrumental variables to estimate the effect of an exposure on an outcome. We performed UVMR in 2 steps (2-step MR) to estimate the contribution of potential causal mediators, followed by multivariable MR (MVMR) (33), mediation analysis (34), and sensitivity analysis. The variants used for MR in each trait are summarized in Tables ST1–ST3. At each stage of 2-step MR and MVMR, only Steiger-filtered single nucleotide polymorphisms (SNPs) were used (see Supplemental Methods for more details).

### UK Biobank

UK Biobank received ethical approval from the North-West Multi-Centre Research Ethics Committee (REC reference: 11/NW/03820). All participants gave written informed consent in accordance with the principles of the Declaration of Helsinki. We applied to UKB and had access to the UKB resource (Application No. 103356).

Finally, to complement our 2-sample UVMR analysis and to estimate sex-specific causal effects, we also performed a 1-sample UVMR in males and females separately using individual-level data from the UKB (see the Supplement for more details).

#### UKB Outcomes

We defined a variety of outcomes in the UKB, such as T2D, general practitioner (GP)-recorded depression, and depression identified through the first UKB mental health questionnaire (MHQ), as well as lifestyle and socioeconomic factors, including educational attainment, Townsend deprivation index (TDI), cigarettes per day, alcohol consumption (units per day), accelerometry-derived physical activity, and sedentary time. We also created a genetic risk score (GRS) for all the abovementioned traits to use in 1-sample MR (more details are provided in Supplemental Methods).

#### Statistical Analysis

All statistical analyses in the UKB were conducted using a 1-sample MR framework and analysis was done only in unrelated European individuals (*n* ∼ 374,900) (more details are provided in Supplemental Methods). As with UVMR, the MVMR analyses were also performed on the UKB data.

#### Statistical Analysis: Multiple Testing

We used the Benjamini-Hochberg or false discovery rate (FDR) procedure to control the FDR (FDR threshold = .05), as both the mental health phenotypes and lifestyle and socioeconomic traits are highly correlated and therefore Bonferroni correction would be overly conservative.

#### Statistical Analysis: Software Packages

Partial LDSC, Genomic SEM, 2-step MR, mediation analysis, and 2-sample MR analyses were performed using R version 4.1.2, while analyses in the UKB were performed in RStudio version 4.1.1 on the UKB RAP system.

## Results

### Lifestyle and Socioeconomic Factors Were Strongly Genetically Correlated With Both MDD and T2D

There were strong positive genetic correlations of MDD with T2D (*r*_*g*_ = 0.184, 95% CI [0.156 to 0.212]), cigarettes per day (*r*_*g*_ = 0.282, 95% CI [0.224 to 0.340]) and higher poverty (*r*_*g*_ = 0.371, 95% CI [0.405 to 0.337]), and negative correlations were observed for educational attainment (*r*_*g*_ = −0.293, 95% CI [−0.328 to −0.259]), physical activity (*r*_*g*_ = −0.171, 95% CI [−0.219 to −0.123]), and drinks per week (*r*_*g*_ = −0.083, 95% CI [−0.123 to −0.043]). No correlation was observed with sedentary time (Table ST4).

T2D was positively genetically correlated with cigarettes per day (*r*_*g*_ = 0.295, 95% CI [0.233 to 0.356]), drinks per week (*r*_*g*_ = 0.093, 95% CI [0.055 to 0.131]), sedentary time (*r*_*g*_ = 0.192, 95% CI [0.141 to 0.243]) and poverty (*r*_*g*_ = 0.204, 95% CI [0.236 to 0.172]) and negatively correlated with physical activity (*r*_*g*_ = −0.262, 95% CI [−0.305 to −0.218]) and educational attainment (*r*_*g*_ = −0.263, 95% CI [−0.296 to −0.231]) (Table ST4). All the *p* values remained significant after correction for multiple testing. SNP heritability for each phenotype is provided in Table ST5.

### Five Lifestyle and Socioeconomic Factors Influence the MDD-T2D Genetic Correlation

Partial LDSC demonstrated that 5 of the 6 factors altered the MDD-T2D genetic correlation (Figure 1 and Table 1). The MDD-T2D genetic correlation was substantially attenuated when accounting for cigarettes per day (partial *r*_*g*_ = 0.098, 95% CI [0.061 to 0.135]), physical activity (partial *r*_*g*_ = 0.141, 95% CI [0.108 to 0.174]), educational attainment (partial *r*_*g*_ = 0.115, 95% CI [0.082 to 0.148]), and poverty (partial *r*_*g*_ = 0.118, 95% CI [0.083 to 0.154]). In contrast, drinks per week tentatively strengthened the correlation (partial *r*_*g*_ = 0.194, 95% CI [0.164 to 0.224]) (Table 1). Sedentary time did not alter the MDD-T2D genetic correlation (Table 1). We observed similar results using Genomic SEM (Figure S1 and Table ST6), although the drinks per week estimate was not replicated.

**Figure 1:**
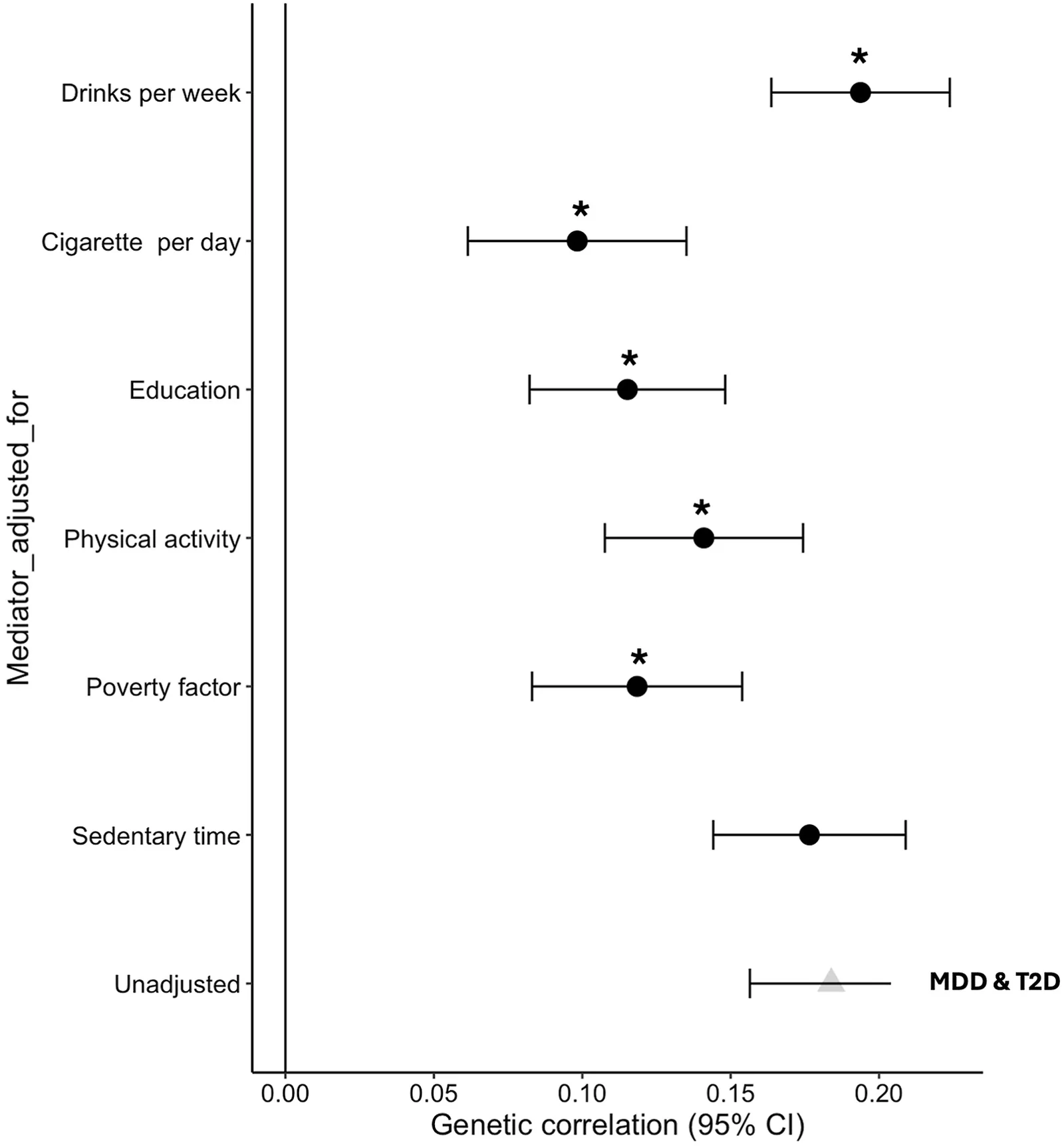
Genetic correlations between MDD and T2D, adjusted for lifestyle and socioeconomic traits using partial linkage disequilibrium score regression: Adjusting for the genetic variations related to all traits (except sedentary time) significantly changes the genetic correlation between MDD and T2D. ∗*p* < .05 signifies that the genetic correlation is significantly different after adjusting for lifestyle/socioeconomic traits compared with the unadjusted MDD-T2D genetic correlation. MDD, major depressive disorder; T2D, type 2 diabetes.

**Table 1:** Results of Partial Linkage Disequilibrium Score Regression After Adjusting for a Range of Traits

Trait 1	Trait 2	Mediator Adjusted for	Partial *r*_*g*_	SE	95% CI	*p* Value^a^	Adjusted *p* Value (FDR/Benjamini-Hochberg)
MDD	T2D	None	0.1839^b^	0.014	0.156–0.211	3.50 × 10^−37^^c^	2.45 × 10^−36^^c^
MDD	T2D	Cigarette per day	0.098	0.019	0.061–0.135	4.76 × 10^−19^^c^	1.11 × 10^−18^^c^
MDD	T2D	Drinks per week	0.194	0.015	0.164–0.224	.0035^c^	.0041^c^
MDD	T2D	Physical activity	0.141	0.017	0.108–0.174	1.76 × 10^−8^^c^	2.46 × 10^−8^^c^
MDD	T2D	Sedentary time	0.177	0.017	0.144–0.209	.1768	.1768
MDD	T2D	Education	0.115	0.017	0.082–0.148	9.71 × 10^−24^^c^	3.40 × 10^−23^^c^
MDD	T2D	Poverty factor	0.118	0.018	0.083–0.154	1.23 × 10^−18^^c^	2.15 × 10^−18^^c^

FDR, false discovery rate; MDD, major depressive disorder; T2D, type 2 diabetes.

a *p* Value represents that the difference between the unadjusted and the adjusted genetic correlation is statistically significant or not.

b Unadjusted genetic correlation between MDD and T2D using LDSC.

c Represents *p* values < .05 for both the unadjusted *p* value from the LDSC analyses and Benjamini-Hochberg FDR controlled *p* values.

### MR Provided Evidence of Causal Relationship of Lifestyle and Socioeconomic Factors With MDD and T2D

#### Step-1 MR

Using Steiger filtered SNPs for UVMR, we demonstrated that MDD was associated with higher cigarettes per day (beta = 0.096, 95% CI [0.074 to 0.119]), higher drinks per week (beta = 0.033, 95% CI [0.015 to 0.051]), and greater poverty (beta = 0.337, 95% CI [0.257 to 0.417]) and lower education (beta = −0.095, 95% CI [−0.121 to −0.069]) (Table 2). There was no evidence of horizontal pleiotropy for cigarettes per day, drinks per week, and poverty, except for education (MR-Egger *p* value = .037), and the results were generally consistent across different pleiotropy-robust methods (Tables ST7 and ST8).

**Table 2:** Two-Step MR, MVMR, and Mediation Analysis Results

Exposure	Mediator	MR Method	No. of SNPs	beta (Raw Values)	SE (Raw Values)	beta^a^	95% CI	*p* Value	Adjusted *p* Value
Two-Step MR: Step 1 (Exposure > Mediator) UVMR Results									
MDD	Cigarette per day	IVW	176	0.1395	0.0165	0.0967	(0.0743 to 0.1191)	2.93 × 10^−17^^b^	3.52 × 10^−16^^b^
	Drinks per week		176	0.0480	0.0130	0.0332	(0.0155 to 0.0509)	2.32 × 10^−4^^b^	6.19 × 10^−4^^b^
	Education		174	−0.1377	0.0195	−0.0954	(−0.1218 to −0.0690)	1.50 × 10^−12^^b^	6.00 × 10^−12^^b^
	Poverty factor		158	0.4870	0.0585	0.3375	(0.2579 to 0.4170)	9.00 × 10^−17^^b^	7.20 × 10^−16^^b^
T2D	Cigarette per day		367	0.0296	0.0062	0.0205	(0.0120 to 0.0289)	2.07 × 10^−6^^b^	6.21 × 10^−6^^b^
	Drinks per week		378	−0.0113	0.0043	−0.0079	(−0.0136 to −0.0021)	7.71 × 10^−3^^b^	1.32 × 10^−2^^b^
	Education		367	−0.0287	0.0060	−0.0199	(−0.0280 to −0.0118)	1.56 × 10^−6^^b^	5.35 × 10^−6^^b^
	Poverty factor		322	0.0688	0.0192	0.0477	(0.0216 to 0.0737)	3.31 × 10^−4^^b^	7.94 × 10^−4^^b^
Two-Step MR: Step 2 (Mediator > Outcome) UVMR Results									
Mediator	Outcome	MR Method	No. of SNPs	beta (Raw Values)	SE (Raw Values)	OR^c^	95% CI	*p* Value	Adjusted *p* Value

Cigarette per Day	MDD	IVW	32	0.0322	0.0453	1.0327	(0.9450 to 1.1285)	4.77 × 10^−1^	5.20 × 10^−1^
Drinks per Week			24	0.1611	0.0962	1.1747	(0.9729 to 1.4184)	.09401665	1.88 × 10^−1^
Education			50	−0.1984	0.0565	0.8200	(0.7341 to 0.9161)	.00044447^b^	1.20 × 10^−3^^b^
Poverty Factor			60	0.0903	0.0149	1.0945	(1.0630 to 1.1268)	1.25 × 10^−9^^b^	1.00 × 10^−8^^b^
Cigarette per Day	T2D		34	0.0893	0.0708	1.0934	(0.9517 to 1.2561)	2.07 × 10^−1^	3.31 × 10^−1^
Drinks per Week			24	−0.5482	0.1666	0.5780	(0.4170 to 0.8011)	.00099635^b^	2.67 × 10^−3^^b^
Education			41	−0.3572	0.0686	0.6997	(0.6117 to 0.8003)	1.90 × 10^−7^^b^	9.12 × 10^−7^^b^
Poverty Factor			65	0.1027	0.0235	1.1082	(1.0583 to 1.1604)	1.22 × 10^−5^^b^	4.88 × 10^−5^^b^
MVMR (Mediator > Outcome Adjusted for Exposure) Results									
Mediator	Exposure Adjusted for	Outcome	No. of SNPs	beta (Raw Values)	SE (Raw Values)	OR^c^	95% CI	*p* Value	Adjusted *p* Value

Education	T2D	MDD	48	−0.2491	0.0389	0.7795	(0.7223 to 0.8413)	1.55 × 10^−10^^b^	2.93 × 10^−10^^b^
Poverty Factor			62	0.0905	0.0114	1.0947	(1.0705 to 1.1195)	2.31 × 10^−15^^b^	9.24 × 10^−15^^b^
Education	MDD	T2D	42	−0.3679	0.0580	0.6922	(0.6178 to 0.7755)	2.20 × 10^−10^^b^	2.93 × 10^−10^^b^
Poverty Factor			65	0.0906	0.0187	1.0949	(1.0554 to 1.1358)	1.27 × 10^−6^^b^	1.27 × 10^−6^^b^
Mediation Analysis									
	Exposure	Outcome	Mediation Method	SE Method	beta, Indirect Effect	SE	95% CI	Proportion of Mediation (Percentage)^d^	

Education	MDD	T2D	Product method (A)	Delta	0.0507	0.0107	(0.0296 to 0.0717)	0.2867 (28.67%)^e^	
Poverty Factor					0.0441	0.0105	(0.0235 to 0.0648)	0.2498 (24.98%)^e^	
Education	T2D	MDD			0.0072	0.0019	(0.0035 to 0.0108)	0.343 (34.3%)^e^	
Poverty Factor					0.0062	0.0019	(0.0025 to 0.0100)	0.2987 (29.87%)^e^	

IVW MR results were used for mediation analysis. Product method A = (Exposure > Mediator, UVMR) × (Mediator > Outcome, MVMR adjusted for exposure). The latent variable in the poverty factor represents the household income, and a negative sign means lower income and higher poverty. Therefore, we switched the negative signs in beta coefficients to positive values for easier interpretations, and now positive value means higher poverty. Adjusted *p* value used FDR/Benjamini-Hochberg.

FDR, false discovery rate; IVW, inverse variance weighted; MDD, major depressive disorder; MR, Mendelian randomization; MVMR, multivariable MR; OR, odds ratio; SNP, single nucleotide polymorphism; T2D, type 2 diabetes; UVMR, univariable MR.

a Representing change in outcome per doubling in exposure genetic liability.

b Represents *p* values < .05 for both the unadjusted *p* value and Benjamini-Hochberg FDR controlled *p* values.

c Representing change in outcome per unit higher genetically instrumented mediator.

d Proportion of mediation was calculated by dividing indirect effect with total effect (exposure > outcome, UVMR).

e Indirect effect is significant and therefore the proportion of mediation is interpretable.

Similarly, a higher genetic liability to T2D was also associated with higher cigarettes per day (beta = 0.020, 95% CI [0.012 to 0.028]), greater poverty (beta = 0.047, 95% CI [0.021 to 0.073]), lower education (beta = −0.019, 95% CI [−0.028 to −0.011]), and fewer drinks per week (beta = −0.007, 95% CI [−0.013 to −0.002]) (Table 2). There was evidence of horizontal pleiotropy for all except drinks per week (MR-Egger *p* value = .393) (Tables ST7 and ST8).

#### Step-2 MR

A higher genetic liability to cigarettes per day was not associated with either MDD or T2D in step 2 of MR. Drinks per week was causally associated with 0.578 lower odds of T2D (95% CI [0.417 to 0.801]); however, no association was observed with MDD (Table 2).

A higher genetic liability to education was also associated with lower odds of MDD (odds ratio [OR] = 0.82, 95% CI [0.734 to 0.916]) and T2D (OR = 0.699, 95% CI [0.611 to 0.800]). Similarly, poverty was also associated with higher odds of MDD (OR = 1.094, 95% CI [1.063 to 1.126]) and T2D (OR = 1.108, 95% CI [1.058 to 1.160]) (Table 2).

There was no evidence of horizontal pleiotropy, except in cigarettes per day to MDD direction (MR-Egger *p* value = .027), and the results were generally consistent across different pleiotropy-robust methods (Tables ST7 and ST8).

### MVMR and Mediation Analysis

Results from 2-step MR suggested that a higher genetic liability to education and poverty are potential mediators, as they demonstrated causal associations at both stages. Therefore, MVMR analysis was performed only for these mediators to examine the direct effect of both the exposure and mediator on the outcome (Table 2 and Table ST7). The causal association of MDD with T2D (OR_unadjusted_ = 1.130) was attenuated when adjusting for education (OR_adjusted_ = 1.089) and the poverty factor (OR_adjusted_ = 1.095) (Table ST7). The direct effect (MVMR adjusted for MDD exposure) of each of these mediators on T2D was almost equivalent to their total effect (from mediator to T2D, UVMR) (Table 2).

In the T2D→MDD direction, the causal effect of T2D on MDD (OR_unadjusted_ = 1.014) was also attenuated to null when adjusted for education (OR_adjusted_ = 1.008) and poverty factor (OR_adjusted_ = 1.010) (Table ST7). The direct effect (MVMR adjusted for T2D exposure) of both these mediators on MDD was comparable to their total effect (mediator to MDD, UVMR) (Table 2).

To quantify the indirect effect, we primarily used product method A (see Supplemental Methods) to report our findings (Table 2) as it allows us to estimate the direct effect of the mediator on the outcome conditioned on exposure.

The proportion of mediation analysis (indirect effect/total effect [exposure to outcome UVMR]) showed that education and poverty explained 28.67% and 24.98% of the total effect of MDD on T2D, respectively (Figure 2 and Table 2).

**Figure 2:**
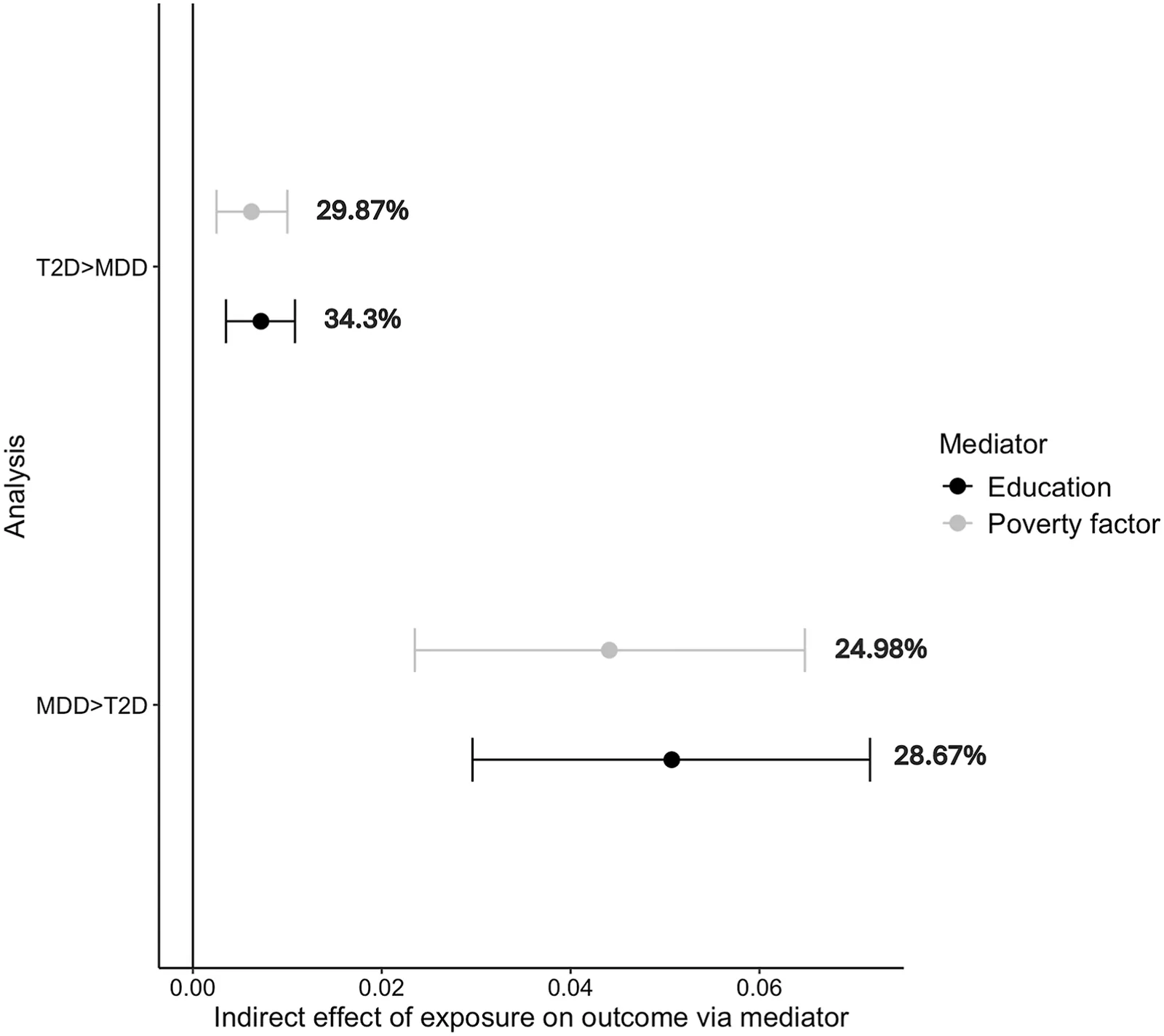
Results of mediation analysis showing indirect effect of (lower part) MDD on T2D via education and poverty factor; (upper part) T2D on MDD via education and poverty factor. Note: Proportion of mediation via each of these factors are given next to error bars. MDD, major depressive disorder; T2D, type 2 diabetes.

In the T2D→MDD direction, there was robust evidence that education and poverty mediated 34.3% and 29.87% of the total effect of T2D on MDD, respectively (Table 2).

Indirect effects were also calculated using product method B, with comparable results (Table ST7).

### Sensitivity Analysis Strengthened the Evidence Suggesting the Importance of Education and Poverty

When additionally adjusting for the genetic effect of education in the MDD→T2D direction, only the indirect effect of the poverty factor decreases from 24.98% mediation to 2.03% mediation, and no change was observed for mediation by education when adjusted for poverty (unadjusted mediation: 28.67%, adjusted mediation: 28.57%) (Table ST9). In the opposite direction, adjusting for education decreases the proportion of mediation by poverty from 29.87% to 21.39%. However, adjusting for the genetics of the poverty factor attenuates the indirect effect of education to null (Table ST9).

#### Replication of 2-Step MR Results in Individual-Level Data in UKB

One-sample MR in UKB broadly supported step 1 of the 2-step MR findings. For example, a doubling in MDD genetic liability (GP-derived MDD) associated with increased smoking and alcohol use, fewer years of education, and higher deprivation (Figure S2 and Table ST10). MR in the opposite direction was also broadly consistent with step 2 of the 2-step MR results, where genetically instrumented educational years and TDI were associated with 0.917 lower and 1.311 higher MDD odds, respectively, in all individuals (Figure S3 and Table ST10).

A doubling in T2D genetic liability robustly associated with lower alcohol units per day, fewer years in education, lower moderate-vigorous physical activity, and higher deprivation (Table ST10). There was limited or no evidence for a genetic liability of T2D being causally associated with cigarettes per day, sedentary time, and light physical activity (Figure S4 and Table ST10). Genetically predicted light and moderate vigorous physical activity (actigraphy derived) causally associated with lower T2D odds (Table ST10). Similarly, genetically instrumented longer educational duration associated with 0.872 lower T2D odds, while the number of alcohol units per day and higher TDI associated with 1.080 and 1.761 higher odds of T2D, respectively (Figure S5 and Table ST10).

All associations survived correction for multiple testing, and the results were broadly consistent in sex-stratified analyses, with men and women showing effects in the same direction and of similar magnitude (Table ST10).

We also performed MVMR in UKB data to see if there is any change in the MDD-T2D causal relationship when conditioned on socioeconomic factors. We observed clear attenuation of the causal association of MDD (GP derived) with T2D and vice versa when adjusted for the genetics of TDI and education (Table ST11).

UVMR was done for actigraphy-derived physical activity and sedentary time measures to examine the general trend for causal association with MDD and T2D, but these variables were not considered for any other analysis. We used a relaxed genome-wide significant (GWS) threshold (*p* < 5 × 10^−6^) for physical activity and sedentary time variants to calculate GRS due to limited SNPs at GWS.

## Discussion

We performed a comprehensive genetic analysis to investigate the role of lifestyle and socioeconomic factors in the MDD-T2D relationship. In previous research, we demonstrated a bidirectional causal relationship between MDD and T2D and highlighted the role of insulin resistance in this association (5). Our study builds on prior work and strengthens the evidence by applying multiple genetic epidemiological approaches, demonstrating that the genetics of socioeconomic traits partially mediates the causal relationship between MDD and T2D.

Our collective results from both 2-step MR and UKB data support previous observational (35,36) and genetic (35,37) studies, showing that depression influences both education and poverty and vice versa. We observed attenuation in the MDD-T2D relationship when accounting for genetic variations related to education and poverty using both partial LDSC and MVMR analyses. A substantial proportion of this relationship was found to be mediated via education (28.67%) and poverty (24.98%) genetics.

When the genetic variations related to both education and poverty were modeled together in our multivariable analysis, the mediation by education in this relationship remained, whereas the contribution of poverty was no longer evident. This suggests that the impact of education is more central and somewhat independent of poverty in how MDD genetic liability modulates T2D risk. Our results complement previous longitudinal studies suggesting that education was an important mediator of the MDD-T2D relationship (22,38). Studies have shown that T2D risk is higher among depressed individuals with fewer years of education, as adolescents with depression are more likely to leave education early (22,39), and remaining in school is causally associated with a lower risk of diabetes and mortality (40). Given that depression often emerges during adolescence and early adulthood, it is plausible that depressive episodes disrupt educational progress, thus limiting income potential and contributing to unhealthy behaviors and adverse health outcomes later in life (41). Additionally, depression has been found to impact neurocognitive functioning in young adults, impairing education, vocational attainment, and social functioning (38,42).

Similarly, our analysis shows that there is a bidirectional causal relationship between T2D and both education and poverty. Lower education and income levels have been associated with higher psychological stress and depression in adults (35,43,44), with better education suggested to lead to better jobs, a safer work environment, and better access to health care, ultimately resulting in better health outcomes (45). Our sensitivity analysis further demonstrated that in the T2D→MDD direction, the mediation effect of poverty genetics is independent of education, while mediation via education might be dependent on income factors. It has been shown that household income but not education was associated with major depressive episodes in adults in the United States (46). Additionally, in individuals with diabetes, income but not education was inversely associated with glycated hemoglobin (HbA1c) (47), while in individuals without T2D, education but not income was inversely associated with HbA1c (47), suggesting that socioeconomic factors may interact differently with disease onset and management. Education may delay T2D onset; however, effective management of T2D may require financial flexibility (48). Lower income is associated with a higher risk of depression, cardiovascular risk, and mortality in individuals with T2D (49, 50, 51, 52) and may interfere with long-term T2D management, resulting in adverse health consequences (47,51). This may explain why the proxy measures of poverty using genetic variants emerge as the primary mediator among the socioeconomic factors examined in the T2D→MDD direction.

In the context of our analysis, it is important to highlight that the genetics of education and poverty are highly complex, as they have substantial overlap, yet they exert independent effects (53,54). This is further complicated by cognitive ability which has been found to weaken the association between poverty and mental illness in MR studies, suggesting the multifaceted role of socioeconomic factors (21). Additionally, our MR results have shown that education and poverty have bidirectional relationships with both MDD and T2D, making them potential confounders (a trait affecting both exposure and outcome), colliders (exposure and outcome both affecting the same trait), as well as mediators (exposure to outcome pathway only via a third trait) (Figure S6). MR reduces such bias when its assumptions of independence and exclusion restriction are held. Although our estimates should be interpreted as upper-bound estimates conditional on partially untestable assumptions, particularly given that MR itself is not immune to collider bias in MVMR settings. However, the directionality and consistency of findings across multiple MR and mediation approaches suggest that collider bias is unlikely to fully explain the observed associations.

A bidirectional relationship has been observed between depression and alcohol use, with some sex-specific differences (11,55, 56, 57). However, our results only provided evidence for a higher MDD genetic liability associating with higher drinks per week. The evidence for alcohol consumption on T2D risk is less clear, with some studies suggesting either no (58) or an inverse (59,60) association with T2D, with other studies indicating higher risk (61). The observed reduction in T2D risk due to alcohol intake in our 2-step MR results may be partially explained by increased insulin sensitivity (22,39). Although the literature is insufficient and inconsistent (62), it also highlights potential harmful effects of alcohol consumption, warranting careful interpretation of results (62,63). Our results in UKB data also demonstrated opposite results, showing that genetically instrumented alcohol (units per day) associated with a higher risk of T2D.

Cigarettes per day produced the greatest attenuation of the MDD-T2D genetic correlation in partial LDSC analyses, but its role as a mediator was not supported as it failed to show the causal associations at both stages of the 2-step MR. This discrepancy likely arises because partial LDSC captures shared genetic architecture, whereas MR assesses causal pathways. Given the well-recognized pleiotropic nature of smoking-related phenotypes and the associated genetic variants (64), cigarettes per day may account for part of the genetic overlap between MDD and T2D without representing a direct causal mediator of their relationship. Previous observational and genetic studies have suggested that cigarette smoking is associated with depression (65, 66, 67) and T2D (68) risk, although others have shown inconsistent or no association (58,68). Additionally, smoking behavior is multidimensional, and measures such as smoking status, initiation, heaviness, duration, cessation, pack years, and lifetime smoking each capture different aspects of smoking exposure, with some being more informative than others. Therefore, the present results should be interpreted in the context of the specific smoking measure used.

In UKB data, we found a higher genetic liability to both MDD and T2D associated with lower actigraphy-derived physical activity and higher sedentary time. In the opposite direction, only genetically instrumented higher physical activity associated with lower MDD and T2D risk. Our results align with other MR and cross-sectional studies suggesting that higher physical activity is associated with lower T2D (69, 70, 71) and MDD (14,72) risk, with the protective role on T2D potentially partially mediated via improved fasting insulin levels (64). These results should be interpreted with caution, as we have used a relaxed threshold for instrument selection to see the direction of causal associations and hence are prone to weak instrument bias and inclusion of non-causal variants.

This study had several strengths. Firstly, we used the largest available GWAS data for T2D, MDD, and the lifestyle and socioeconomic traits considered. Secondly, we used UKB individual-level data and validated definitions of mental health outcomes (73,74), which, unlike meta-analysis results, give us homogeneity of definitions, an issue that is particularly important in mental health research. Thirdly, we used several complementary genetic methods to assess the role of lifestyle and socioeconomic factors on the MDD→T2D relationship, including genomic SEM, partial LDSC, MVMR, and 2-step MR methods (75).

We acknowledge several limitations. First, our findings are not fully representative of the general population. The largest available GWAS for most of the lifestyle and socioeconomic traits included in this study were conducted in individuals of European ancestry; therefore, the extent to which these results generalize to other populations remains uncertain. Comparable GWAS of sufficient size and with harmonized phenotypes are currently limited in other ancestries, preventing formal replication and cross-ancestry comparisons. Additionally, although depression is more prevalent in women than in men, our sex-stratified analyses for different traits in UKB found limited evidence of sex-specific differences. However, these analyses were likely underpowered, particularly for detecting modest effects. Future studies using larger, sex-stratified GWASs from diverse population cohorts, such as Our Future Health and the All of Us Research Program, are needed to validate these findings and investigate potential sex- and ancestry-specific genetic effects in more detail. Second, our UKB findings are not generalizable to the population as the UKB is not population representative, with overrepresentation of females and individuals with higher socioeconomic status (76) and participation bias within the MHQ (77). Third, within the UKB, we used 2 different depression definitions derived from the MHQ and GP records. While our results were generally consistent, they tended to be stronger with GP-derived depression. This may reflect that the GP-derived metric more accurately captures MDD than MHQ data.

Fourth, our findings suggest that education and poverty may act as mediators of the MDD-T2D relationship, although they could also function as colliders or confounders. However, the consistency of the findings across multiple analytical approaches supports their role as partial mediators. Education and poverty were also highly genetically correlated (*r*_*g*_ = 0.84; data not shown) and are influenced by complex social- and population-level factors, making them particularly susceptible to residual population stratification and related biases. Although we further applied several sensitivity analyses to minimize the impact of horizontal pleiotropy, these biases cannot be completely excluded. We observed evidence of horizontal pleiotropy for the T2D→education and T2D→poverty directions based on the MR-Egger intercept. Nevertheless, the direction and statistical significance of these associations were broadly consistent across pleiotropy-robust MR methods, providing some reassurance regarding the underlying relationship. Therefore, the estimated mediation proportions involving education and poverty should be interpreted cautiously, as residual pleiotropy or collider bias in MVMR may have influenced the magnitude of the indirect effects. Finally, we used TDI and units per week phenotypes in the UKB as a proxy measure of the poverty factor and drinks per week phenotypes, respectively, used in the 2-step MR analysis. These phenotypes in the UKB strongly associated with the corresponding GRS created using the GWS SNPs identified in the discovery GWAS, but these results should be interpreted accordingly.

### Conclusions

Our findings suggest a pivotal role for socioeconomic factors, particularly education and poverty, in mediating the bidirectional relationship between MDD and T2D. Genetic predisposition to education appeared to exert a more independent influence on the MDD→T2D pathway, while poverty played a stronger role in the T2D→MDD direction, likely reflecting differences between disease onset and management. Overall, using genetic data, we demonstrated that socioeconomic factors remained significant mediators, displaying complex interrelationships both among themselves and with MDD and T2D. However, genetic liability reflects inherited predisposition rather than direct measurements of behaviors or clinical traits and therefore does not translate directly into real-world risk. Genetic effects capture population-level genetic architecture rather than individual-level causal effects and therefore should not be interpreted as direct predictions for individual behavior or disease risk. Epidemiological studies can complement our research by using direct measures of these traits in real-world settings and evaluating how genetic findings improve our understanding of complex traits within specific social and environmental contexts. Future work should also be focused on disentangling the interrelated effects of education, income, and cognitive ability and integrating gene-environment interactions to clearly understand their roles in the MDD-T2D relationship. Policies and interventions that address broader social constructs, such as improving access to education and reducing poverty, may help reduce overall risk at the population level.

## Supplementary Material

Supplemental Methods and Table SM1

Tables ST1–ST12

Key Resources Table
