# Supplementary material for: Using Genetics to Understand the Role of Lifestyle and Socioeconomic Factors in the Relationship Between Major Depressive Disorder and Type 2 Diabetes
Source: Biol Psychiatry Glob Open Sci. 2026 Sep 9;7(1):100822. doi: 10.1016/j.bpsgos.2026.100822 (PMC13742398; doi:10.1016/j.bpsgos.2026.100822)
Supplement: Supplementary file 1 [file mmc1.pdf]

SUPPLEMENTARY INFORMATION

Using Genetics to Understand the Role of Lifestyle and Socioeconomic Factors in the Relationship Between Major Depressive Disorder and Type 2 Diabetes

Bala *et al.*

Supplementary methods

Genomic Structural Equation Modelling (SEM)

An alternative approach to partial LDSC for investigating shared genetic architecture is Genomic SEM (1). We applied Genomic SEM alongside partial LDSC to examine whether any of the six lifestyle and socio-economic traits influenced the genetic correlation between MDD and T2D. Specifically, we estimated the genetic correlation between MDD and T2D before and after accounting for each lifestyle or socio-economic trait.

Rather than fitting a common factor model to identify a latent genetic factor shared across all traits, we specified our own models to evaluate the contribution of each individual trait. For example, to assess the influence of educational attainment on the shared genetic architecture between MDD and T2D, we specified the following model:

my_model <- 'MDD~ education + T2D~education'

This approach allowed us to determine whether the genetic component of each lifestyle or socio-economic trait explained part of the shared genetic architecture between MDD and T2D. More details about this method can be found on https://github.com/GenomicSEM/GenomicSEM.

MR Analyses

MR relies on several assumptions:

1. The exposure SNPs are robustly associated with the measured exposure. This is quantified by the F-statistic, which can be approximated by the ratio of the SNP-exposure association estimate, $\hat{\beta}$, and its standard error, $SE(\hat{\beta})$, squared (equation 1) (2). As a general rule an F-statistic of >10 indicates that the level of weak instrument bias is likely to be small (3).

   Equation 1 $$F = \left(\frac{\hat{\beta}}{SE(\hat{\beta})}\right)^2$$

   F-statistics were calculated for our exposure variants and reported in supplementary tables 1-3.
2. The exposure SNPs are not associated with confounding factors that bias conventional epidemiological associations.
3. The exposure SNPs are only associated with the outcome through the risk factor.

Two-step MR

Two step MR is a type of 2 sample MR where univariable MR (UVMR) is performed in two steps, Step 1: Exposure to mediator UVMR, and Step 2: mediator to outcome UVMR. This is usually done to identify if exposure-outcome association (either previously established or has supportive evidence from MR) is mediated by a third factor/trait. Exposure SNPs (ST 1-3) for each step were extracted from the respective GWAS summary statistics by clumping at r^2=0.001, p<5×10^{-8}, representing the exposure association with the exposure-trait-SNP. Steiger filtering (explained in detail below) was used to remove potential pleiotropic exposure SNPs. Finally, we used TwosampleMR R package (https://mrcieu.github.io/TwoSampleMR/) to perform the three two-sample MR methods: inverse-variance weighting (IVW), MR-Egger (4) and weighted median (WM) (5). Here, the IVW approach represents our main analyses, with MR-Egger, and WM used as sensitivity analyses to account for unidentified pleiotropy that could bias our results (6). These methods are described in more detail below:

IVW: Horizontal pleiotropy arises when genetic variants associated with exposure of interest independently impact the outcome via another pathway. IVW assumes the absence of horizontal pleiotropy in a fixed effects model or, in the case of a random effects model that acknowledges heterogeneity among causal estimates, it assumes that the correlation between the strength of the association between genetic instruments and the exposure is not linked to the magnitude of pleiotropic effects (the InSIDE assumption). Additionally, the IVW method relies on the assumption that pleiotropic effects average out to zero. If all the IV assumptions are met, then IVW method provides consistent causal estimates.

MR-Egger: Allows more genetic variants to be pleiotropic. In MR-Egger the magnitude of pleiotropic effect is independent of strength of association between genetic instruments and exposure. It also assumes that genetic variants used as IVs should be accurately measured or negligible measurement error should be there. MR-Egger provides precise causal estimates even if IVs violates the exclusion restriction assumption. Pleiotropy can be quantified using the MR-Egger intercept indicating potential bias due to directional pleiotropy if the intercept p-value <0.05. However, when InSIDE assumptions are violated, MR-Egger estimates are less precise and has higher risk of type 1 error.

Weighed Median: less likely to be influenced by the outliers compared to other models. It provides consistent estimates if at least 50% of the weights comes from valid IVs. It does not rely

on a fixed effects assumption and can be applied in the presence of heterogeneity among genetic variants.

Steiger filtering: It is a sensitivity test to identify the validity and directionality of instrumental variables (IVs) used for exposure to outcome MR analysis**.** It assumes that valid IVs should explain more variance in exposure than in outcome and the IVs that do not follow these assumptions are excluded from analysis. We followed this principle and used following two approaches for filtering potential pleiotropic SNPs for two step MR analysis: a) removing SNPs that are at GWS with the outcome traits, b) searching GWAS catalogue (https://www.ebi.ac.uk/gwas/home) for known associations related to exposure SNPs and removing the ones that are at GWS with other traits that may directly or indirectly affect the outcome. Details are given below:

- MDD exposure: SNPs that were associated with T2D, body mass index, waist circumference (WC), hip circumference (HC), metabolic syndrome and insomnia were removed.
- T2D exposure: SNPS related to MDD, insomnia, BMI, metabolic syndrome, Waist hip ratio (WHR), WC, obesity, neuroticism, WC adj BMI, HC adj BMI, and WHR adj BMI were removed.
- Cigarette per day exposure: SNPs related to Anxiety, insomnia, BMI, Schizophrenia, and neuroticism were removed
- Drinks per week exposure: SNPs related to T2D, MDD, BMI, and neuroticism, Schizophrenia were removed
- Education exposure: SNPS related to BMI, T2D, MDD, and insomnia were removed
- Poverty factor exposure: SNPs related to BMI, T2D, and MDD were removed

Multivariable MR (MVMR):

MVMR allows us to estimate the direct effect of either exposure (eg. MDD) on outcome trait (eg. T2D) and vice versa, whilst accounting for the third trait (potential mediator, eg lifestyle and socio-economic traits). A key feature of MVMR is that it estimates the effect of each exposure (here either MDD or T2D and a lifestyle and socio-economic traits) included in the estimation on the outcome conditional on other exposures included in the model and therefore provides the direct rather than total effects for each exposure.

Mediation analysis using Product method:

To quantify and compare indirect effect by mediation analysis, we used two different product methods [A) exposure to mediator UVMR * mediator to outcome MVMR (adjusted for exposure) (collider prone), B) exposure to mediator UVMR * mediator to outcome UVMR (collider robust)]. Each of these methods has its own limitations. Using the MVMR approach in the second step (mediator to outcome, adjusted for the exposure) may introduce collider bias under certain scenarios. In contrast, using method B estimates the total effect of the mediator on the outcome, which may include pathways operating through the exposure or other shared mechanisms and therefore may not isolate the direct mediator effect required for mediation analysis. We primarily used product method A for reporting our findings while acknowledging the collider bias.

UK-biobank recruitment and participants details: The UK Biobank (UKB) is a large-scale cohort comprising over 500,000 participants, aged 37 to 73 at the time of recruitment (2006–2010), and has been thoroughly described in prior publications (7). At baseline, comprehensive phenotypic information was collected through questionnaires, physical assessments, and biological sampling. Participants consented to long-term follow-up via linkage to their electronic health records, including inpatient and general practitioner (GP) data, and to take part in future research assessments. Biological specimens-including blood, urine, and saliva-were collected for downstream analyses. Genotyping was conducted using the Affymetrix Axiom UK Biobank array (~450,000 participants) and the UKBiLEVE array (~50,000 participants), followed by centralized quality control procedures (7). To mitigate the potential impact of genetic confounding from population stratification, analyses were adjusted for the first five genetic principal components. For the present study, we analyzed data from up to 379,430 individuals of unrelated European ancestry, determined via principal component analysis (8), who had genetic information and either GP records or mental health questionnaire data, along with clearly defined type 2 diabetes (T2D) case-control status.

Genetic risk score (GRS) or Polygenic risk score: We constructed genetic risk score (GRS) for MDD, T2D and lifestyle and socio-economic traits using genome-wide significant SNPs identified from their respective GWAS summary statistics (details in method section of main manuscript) (**ST12**)(Note: A relaxed threshold of $p<5*10^{-06}$ was used for physical activity and sedentary time). The GRS were generated using a publicly available custom pipeline (https://lcpilling.github.io/ukbrapR/) which automates SNP selection, harmonization, and score calculation.

To validate the created GRS, we tested their association with the corresponding phenotypes within UKB using regression, adjusting for key covariates (e.g., age, sex, first assessment centre, genotyping chip and first five principal components of ancestry) (Table SM1). Once validated, these GRS were used as instruments in place of individual SNPs to perform one-sample MR analyses in UKB. This approach reduces instrument complexity, increases statistical power, and reduces potential biases arising from weak or pleiotropic individual SNPs, while still capturing the aggregated genetic liability for each trait.

Table SM1: Association of different GRS with respective phenotypes

GRS name	Phenotype in UKB	Effect estimate	S.E.	P-value	N
**MDD**	MDD (Depressed ever)	222.6	6.04	<2e-16***	99644
**MDD**	MDD (GP derived)	118.7	3.597	<2e-16***	169927
**T2D**	T2D	164.1	1.490	<2e-16***	379383
**Sedentary time**	Actigraphy derived sedentary time	4.776	2.790	<2e-16***	81394
**Physical activity**	Actigraphy derived light Physical activity	8.115	0.315	<2e-16***	81394
**Physical activity**	Actigraphy derived moderate-vigorous Physical activity	1.824	0.102	<2e-16***	81394
**Education**	Education years	511.69	11.43	< 2e-16***	375801
**Poverty factor**	TDI	-59.69	2.480	< 2e-16***	378791
**Drinks per week**	Units per day	363.6	8.150	< 2e-16***	320066
**Cigarette per day**	Cigarette per day	1011	6.559	< 2e-16***	27599

UKB derived phenotypes

GP derived MDD

MDD within UKB was coded using the linked GP records. As per the Fabbri et al study (9) cases were defined if they had: a) at least two diagnostic codes for a depressive disorder, at any time point and b) no diagnostic code for bipolar disorder, psychotic disorders or substance abuse related disorders. In unrelated Europeans, we had 23833 cases and 146094 controls.

Mental Health Questionnaire (MHQ) derived MDD (Depressed Ever)

A total of 145,668 participants completed the first UK Biobank Mental Health Questionnaire (MHQ). Depression phenotypes were derived using publicly available R code (https://data.mendeley.com/datasets/kv677c2th4/3), as previously described (10). Depression was assessed using the Composite International Diagnostic Interview Short Form (CIDI-SF) and the Patient Health Questionnaire-9 (PHQ-9). From the CIDI-SF, we generated a binary measure indicating lifetime major depression and a continuous measure reflecting the severity of lifetime depression. For depressed ever MDD phenotype, we had 29563 cases and 70081 controls.

Type 2 diabetes

Type 2 diabetes was derived from multiple sources within the UK Biobank, using data field 130709. Individuals were excluded as a type 2 diabetes case if they were on insulin within the first year. In unrelated Europeans we had 32595 cases and 346788 controls.

Physical activity and Sedentary time (accelerometery derived):

These metrics were derived from the actigraphy devices (Axivity AX3) which was worn by 103,711 individuals from the UK Biobank for up to 7 days. The details of how the data was collected, processed and transformed has been explained in detail in prior publications (11). We had 81394 individuals available for this variable and inverse normalised physical activity and sedentary time metrics was used for downstream analysis.

Education years:

We used UKB data field 6138 to create this phenotype and we had 375812 individuals in this category.

Townsend deprivation Index:

Townsend deprivation index (TDI) was used as a proxy for socio-economic status, which was collected at UK Biobank recruitment in data field 22189. All individuals are assigned a score based on their post code at time of recruitment. We had 378791 individuals in this category.

Note: We have used "Poverty factor" phenotype for 2 sample MR, which was derived from household income, occupational income and social deprivation/TDI from UKB, using a common factor model in Genomic SEM. There is a strong genetic correlation (rg: -0.8007, SE: 0.0344) of "poverty factor" phenotype with social deprivation/Townsend deprivation index (TDI) as shown in the discovery GWAS paper (12). Additionally, GRS for poverty factor is strongly associated with TDI in UKB (Table SM1). Therefore, TDI has been used as a proxy for poverty factor phenotype in 1-sample UVMR in UKB.

Alcohol use (Units per day):

Units per day were derived from UK Biobank self-reported alcohol intake. For weekly drinkers {data fields 1568 (red wine), 1578 (white wine), 1588 (beer/cider), 1598 (spirits), 1608 (fortified wine)}, weekly units were calculated using following standard UK conversion factors:

Weekly units=1.5×(red wine + white wine) +2.8×beer/cider +spirits + fortified wine

Each additional alcoholic drinks (data field 5364) was then added at 1.5 units each, then divided by 7 to obtain units per day.

For monthly drinkers {data field 4407(red wine), 4418 (white wine), 4429 (beer/cider), 4440 (spirits), 4451 (fortified wine), 4462 (additional alcoholic drinks)}, total monthly units were calculated similarly as explained above and divided by 30.4 to get units per day metrics. Participants reporting never drinking (data field 1558 ==6) were assigned 0 units/day. We had 320066 individuals in this category.

Cigarettes per day: Cigarettes per day was derived from various UKB smoking variables. If someone's smoking status (data field 20116) is 0, 1, and 2, they are considered as "never smoker", "current smokers" and "past smokers" respectively. For current smoker, their number of cigarettes per day count was then taken from the self-reported number of cigarettes currently smoked per day (data field 3456). For past smokers, it was taken from the self-reported number of cigarettes previously smoked per day (data field 2887). Never smokers were assigned a value of zero. We had 27599 people for this variable.

References:

1. Grotzinger AD, Rhemtulla M, de Vlaming R, Ritchie SJ, Mallard TT, Hill WD, et al. Genomic structural equation modelling provides insights into the multivariate genetic architecture of complex traits. Nat Hum Behav. 2019;3(5):513-25.
2. Bowden J, Del Greco MF, Minelli C, Zhao Q, Lawlor DA, Sheehan NA, et al. Improving the accuracy of two-sample summary-data Mendelian randomization: moving beyond the NOME assumption. Int J Epidemiol. 2019;48(3):728-42.
3. Sanderson E, Glymour MM, Holmes MV, Kang H, Morrison J, Munafò MR, et al. Mendelian randomization. Nature Reviews Methods Primers. 2022;2(1):6.
4. Bowden J DSG, Burgess S. Mendelian randomization with invalid instruments: effect estimation and bias detection through egger regression. . Int J Epidemiol 2015;44:512–25.
5. Bowden J DSG, Haycock PC, Burgess S. Consistent estimation in Mendelian randomization with some invalid instruments using a weighted median estimator. Genet Epidemiol 2016;40:304–14.
6. Stephen Burgess SGT. Interpreting findings from Mendelian randomization using the MR-Egger method. European Journal of Epidemiology. 2017;32:377–89.
7. Bycroft C, Freeman C, Petkova D, Band G, Elliott LT, Sharp K, et al. The UK Biobank resource with deep phenotyping and genomic data. Nature. 2018;562(7726):203-9.
8. Tyrrell J, Zheng J, Beaumont R, Hinton K, Richardson TG, Wood AR, et al. Genetic predictors of participation in optional components of UK Biobank. Nature communications. 2021;12(1):886.
9. Fabbri C, Hagenaars SP, John C, Williams AT, Shrine N, Moles L, et al. Genetic and clinical characteristics of treatment-resistant depression using primary care records in two UK cohorts. Molecular Psychiatry. 2021;26(7):3363-73.
10. Davis KAS, Coleman JRI, Adams M, Allen N, Breen G, Cullen B, et al. Mental health in UK Biobank - development, implementation and results from an online questionnaire completed by 157 366 participants: a reanalysis. BJPsych Open. 2020;6(2):e18.
11. Doherty A, Jackson D, Hammerla N, Plötz T, Olivier P, Granat MH, et al. Large scale population assessment of physical activity using wrist worn accelerometers: the UK biobank study. PloS one. 2017;12(2):e0169649.
12. Marchi M, Alkema A, Xia C, Thio CH, Chen L-Y, Schalkwijk W, et al. Investigating the impact of poverty on mental illness in the UK Biobank using Mendelian randomization. Nature Human Behaviour. 2024;8(9):1771-83.

Supplementary figures

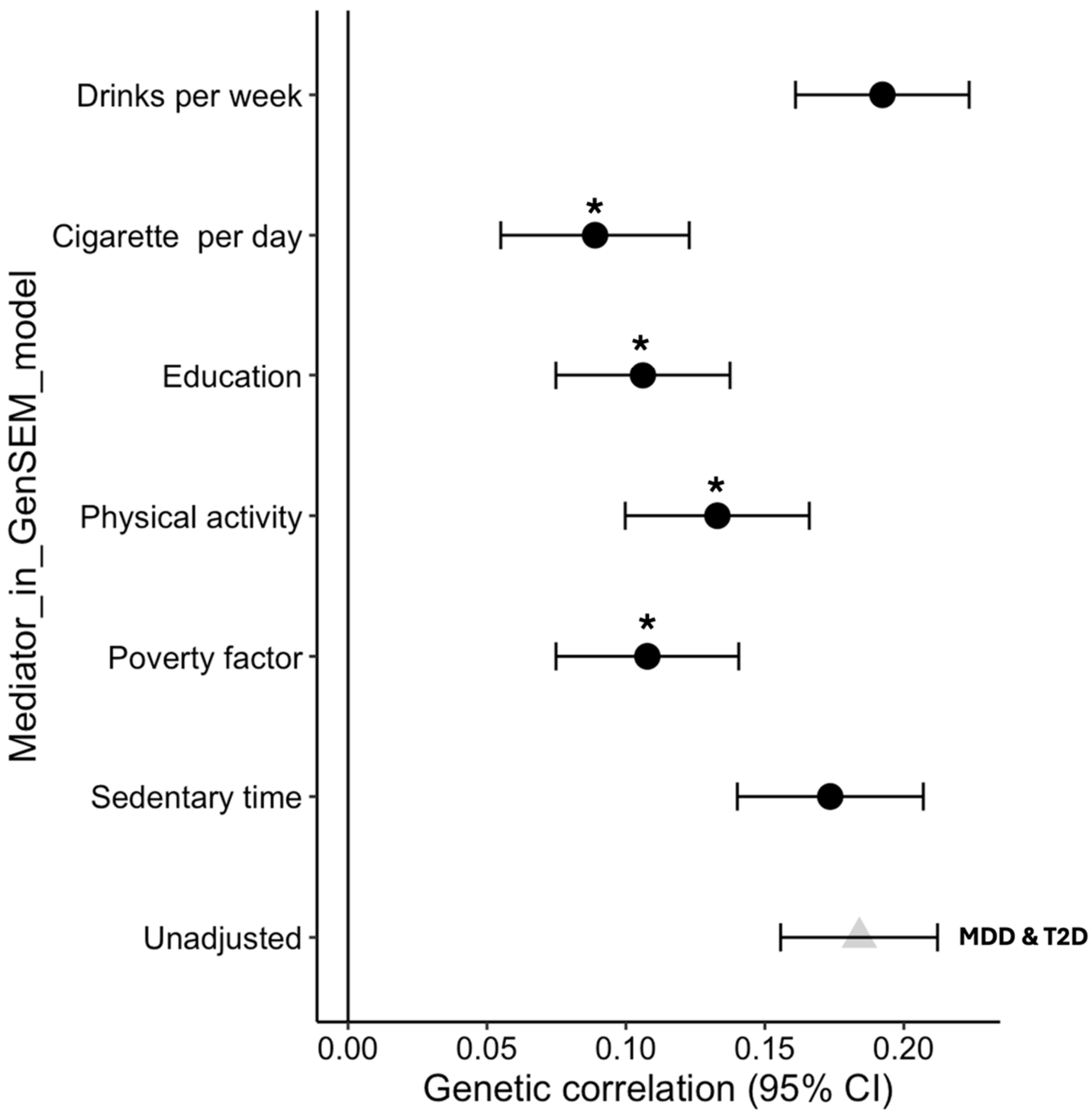

Fig S1: Genomic Structural equation modelling results show that adjusting for all lifestyle and socio-economic traits except sedentary time significantly attenuates the genetic correlation between MDD and T2D. *p<0.05 after adjusting for mediator in Genomic SEM model. (Abbreviations used, MDD: Major depressive disorder, T2D: type 2 diabetes, GenSEM: Genomic Structural equational modelling)

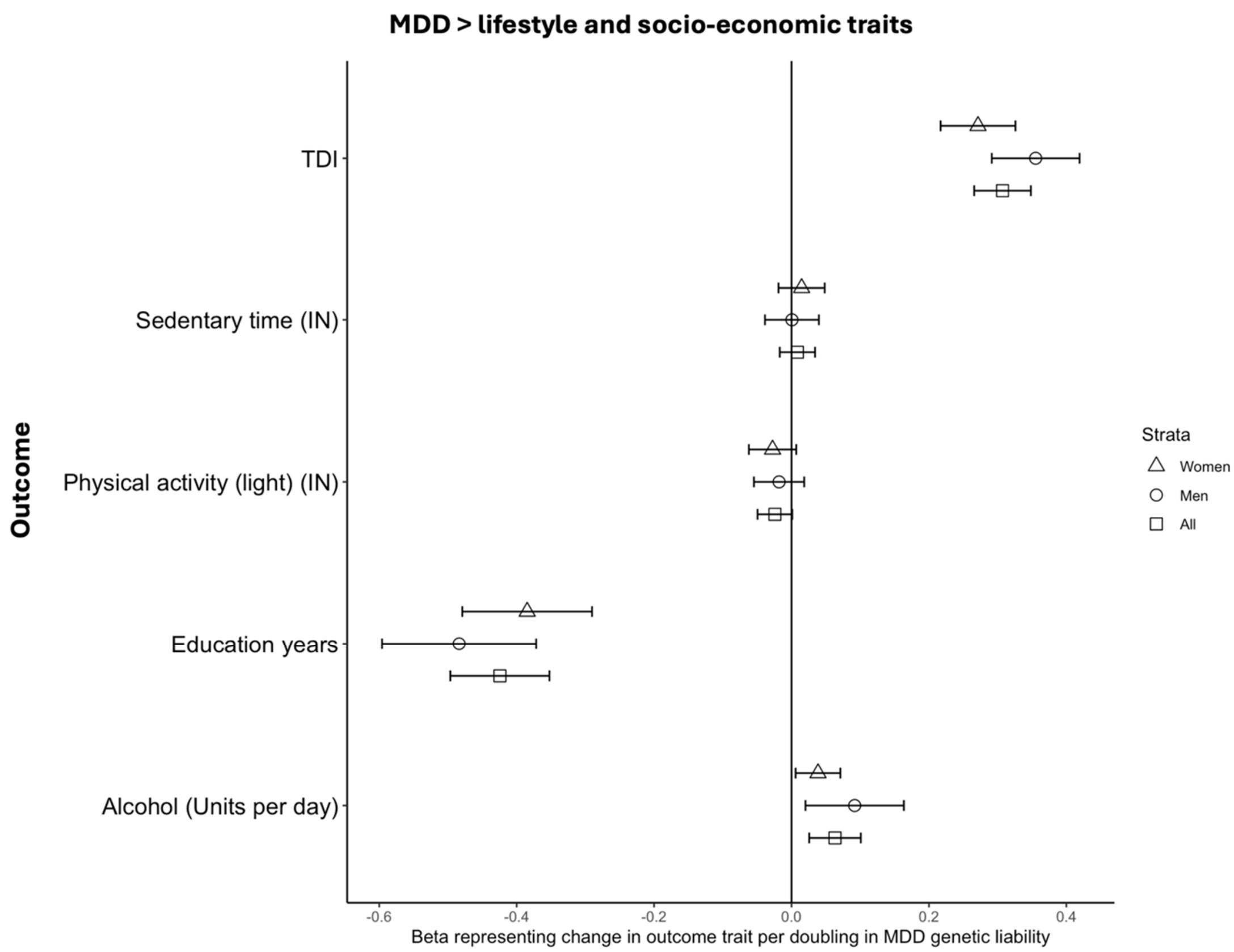

Fig S2: Sex stratified UVMR in UK-biobank (UKB) shows increase in townsend deprivation index or poverty (TDI), alcohol consumption (units per day) and decrease in overall education years per doubling in MDD (GP derived) genetic risk. (Abbreviations used, UVMR: univariable Mendelian randomization, GP: general practitioner, GRS: genetic risk score). Note: GRS of MDD was used as a proxy of exposure genetic instruments for analysis and results for cigarette per day has not been shown due to large confidence intervals.

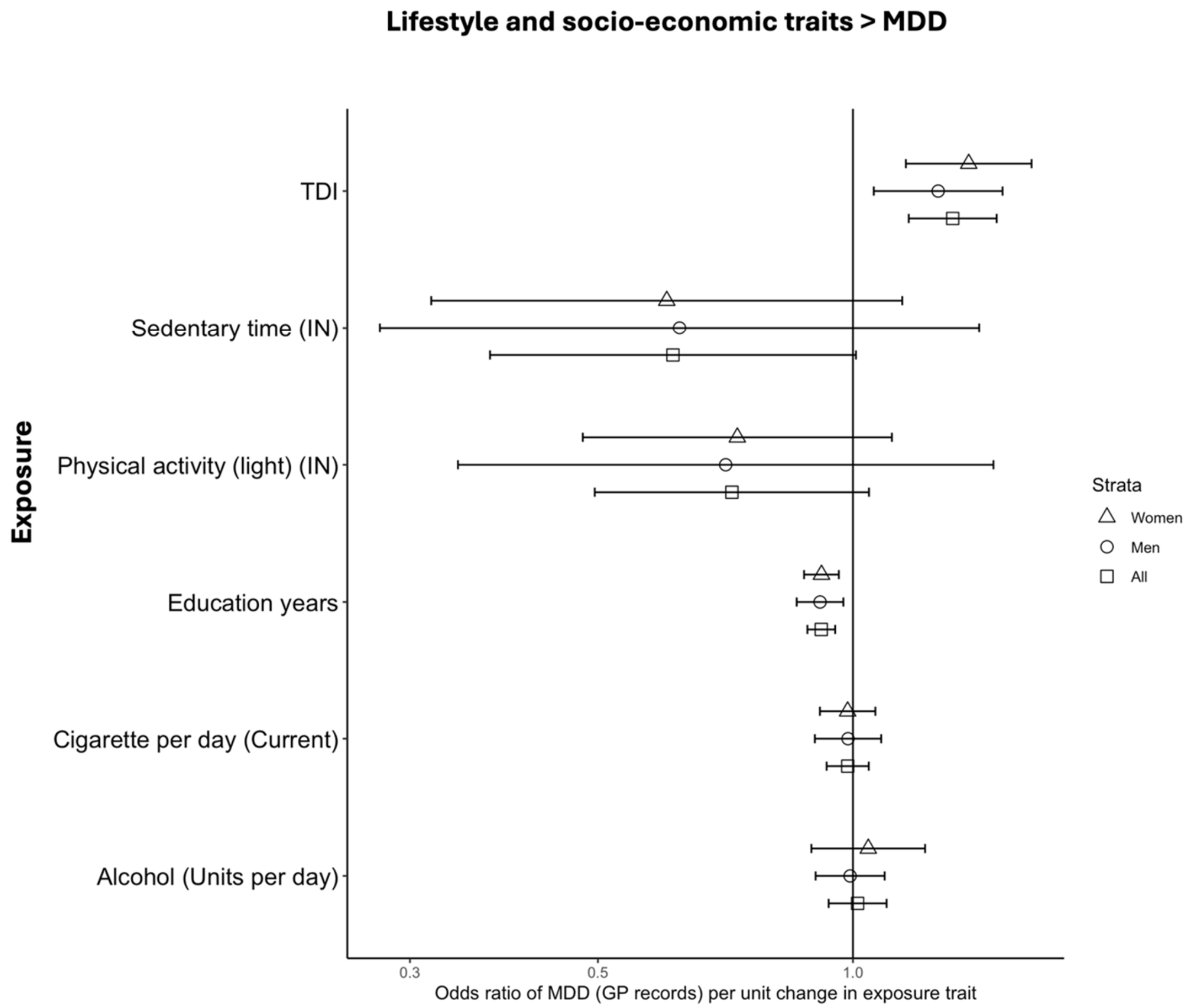

Fig S3: Sex stratified UVMR in UK-biobank (UKB) shows that per unit increase in TDI (townsend deprivation index) is causal for increased MDD risk, while education years is causal for decrease in MDD risk. (Abbreviations used, UVMR: univariable Mendelian randomization, GP: general practitioner, GRS: genetic risk score). Note: GRS of exposure traits was used as a proxy of genetic instruments for analysis.

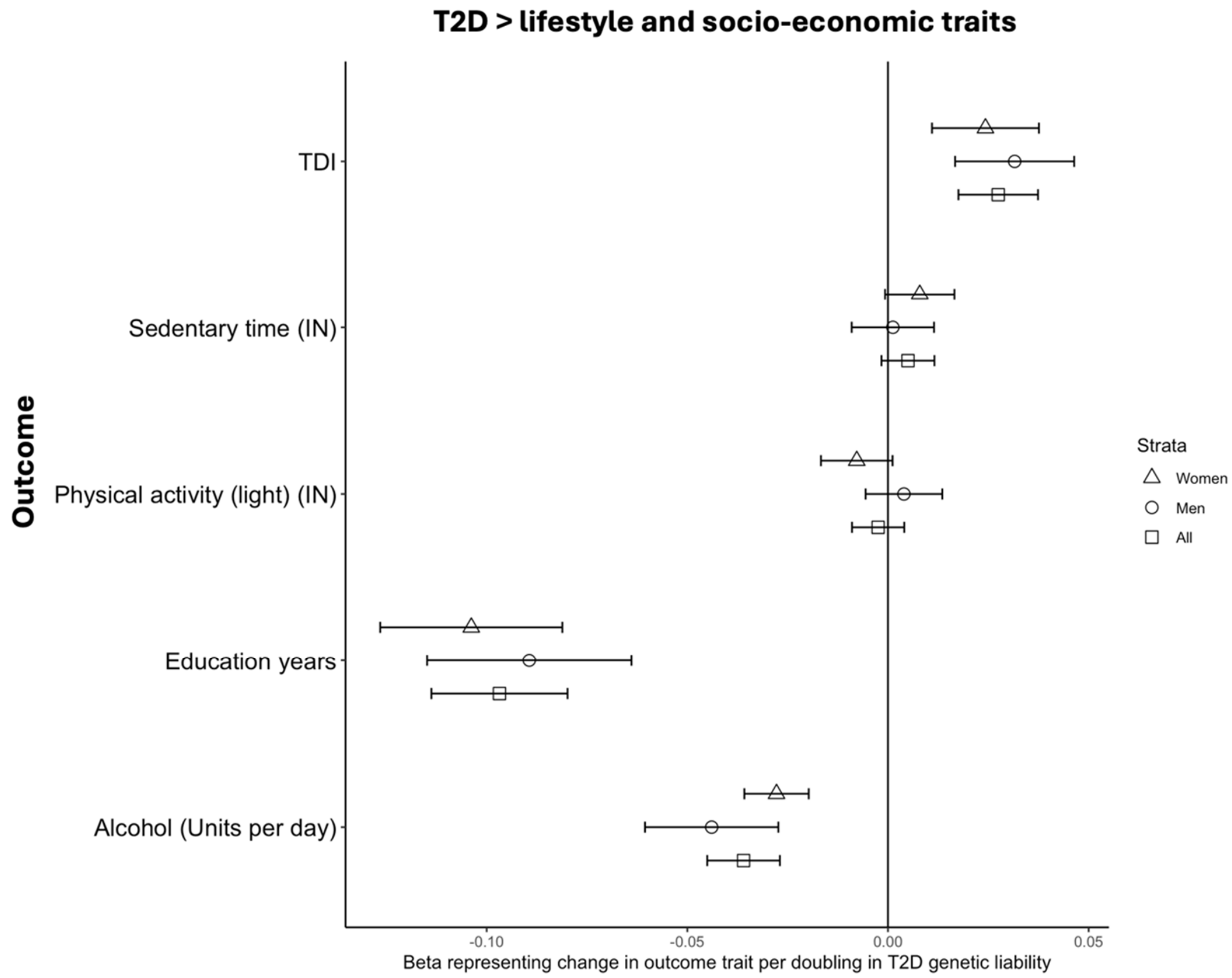

Fig S4: Sex stratified UVMR in UK-biobank (UKB) shows increase in poverty or townsend deprivation index (TDI) and decrease in alcohol consumption (units per day) and education years per doubling in T2D genetic risk. (Abbreviations used, UVMR: univariable Mendelian randomization, GRS: genetic risk score). Note: GRS of T2D was used as a proxy of exposure genetic instruments for analysis and results for cigarette per day has not been shown due to large confidence intervals.

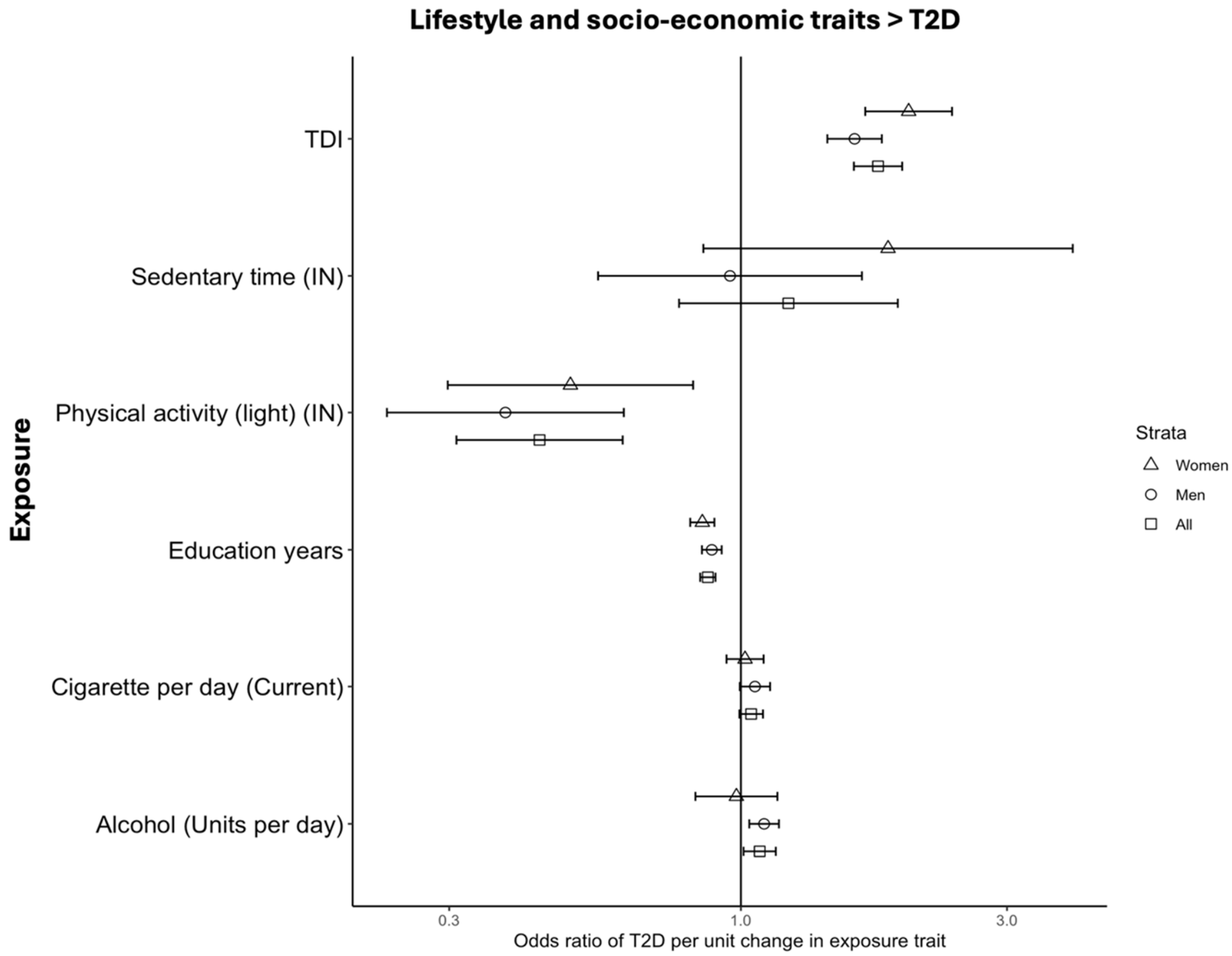

Fig S5: UVMR in UK-biobank (UKB) shows that per unit increase in TDI (townsend deprivation index) is causal for increased T2D risk, while more education years and physical activity (light) is causal for decreased T2D risk in sex stratified analysis. (Abbreviations used, UVMR: univariable Mendelian randomization, GRS: genetic risk score). Note: GRS of exposure traits was used as a proxy of genetic instruments for analysis.

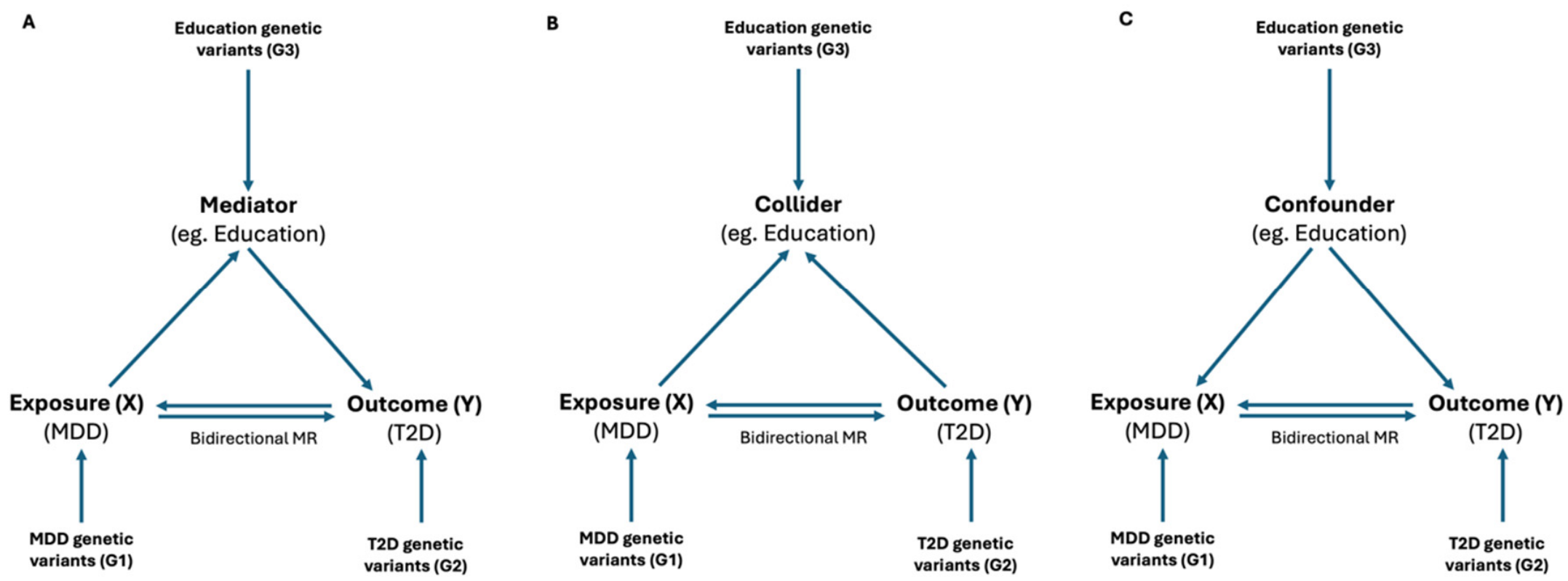

Fig S6: Representative diagram showing the complex relationship between MDD, T2D and socio-economic factors (eg. Education). There is a bidirectional relationship between MDD and T2D, education is associated with both MDD and T2D (A) and in-turn both MDD and T2D are also associated with education (B), making it a potential confounder, collider as well as a mediator (C).
